# Supplementary material for: Model design choices impact biological insight: Unpacking the broad landscape of spatial-temporal model development decisions
Source: PLoS Comput Biol. 2024 Mar 8;20(3):e1011917. doi: 10.1371/journal.pcbi.1011917 (PMC10954156; doi:10.1371/journal.pcbi.1011917)

S2 Fig. **System representation emergent behavior**. *Related to Fig 2.* (**A**) Time course of emergent metrics for different choices of dimension, grouped by geometry. (**B**) Time course of emergent metrics for different choices of geometry, grouped by dimension.

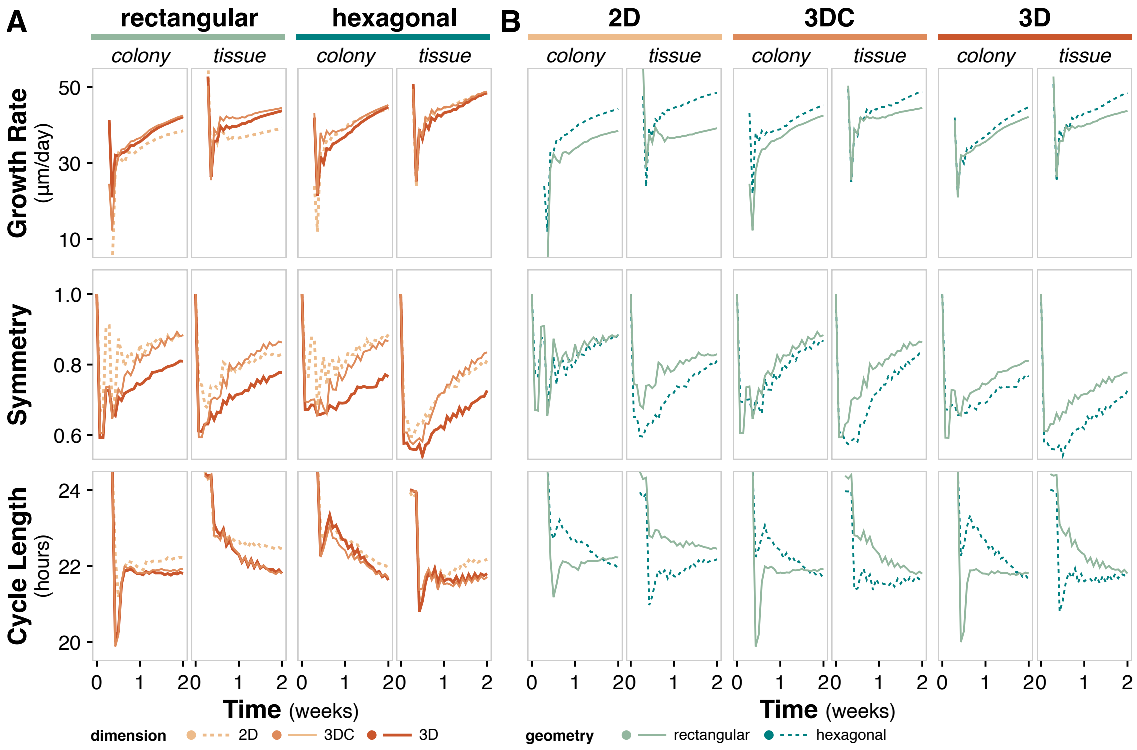

Supplement: S2 Fig — (PDF) [file pcbi.1011917.s002.pdf]
