# Supplementary material for: Model design choices impact biological insight: Unpacking the broad landscape of spatial-temporal model development decisions
Source: PLoS Comput Biol. 2024 Mar 8;20(3):e1011917. doi: 10.1371/journal.pcbi.1011917 (PMC10954156; doi:10.1371/journal.pcbi.1011917)

S3 Fig. **Cell variability simulations**. *Related to Fig 3.* (**A**) Outlines of colonies averaged across replicates. (**B**) Distribution of cell states at t = 15 days for a single replicate.

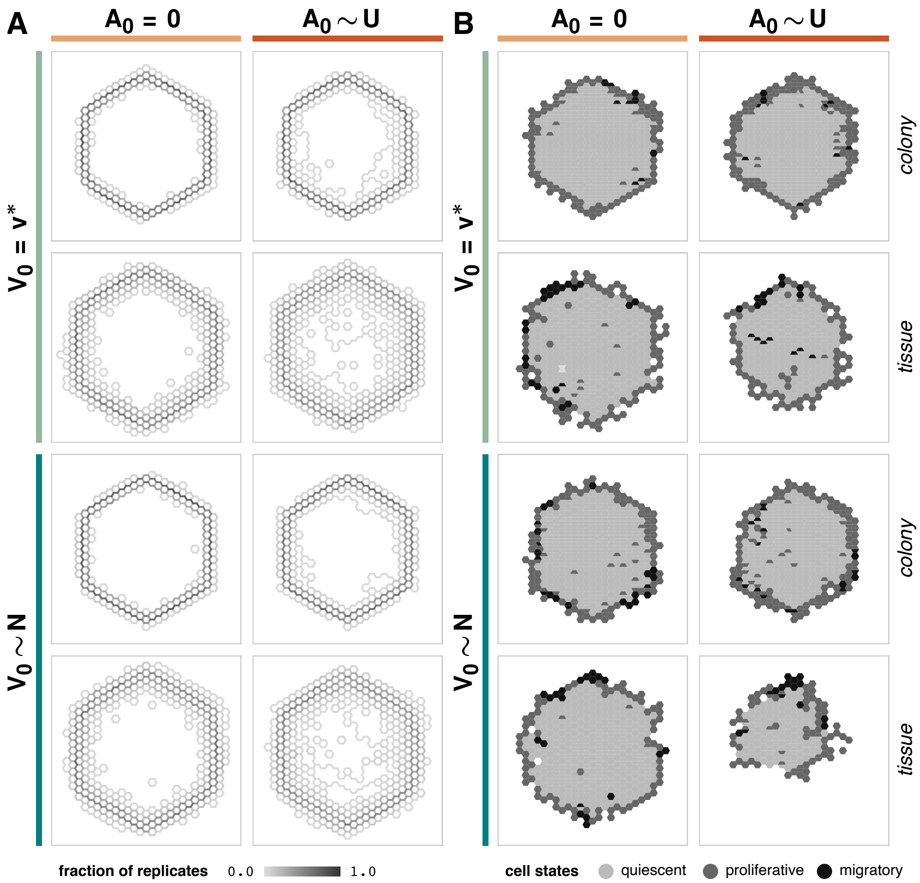

Supplement: S3 Fig — (PDF) [file pcbi.1011917.s003.pdf]
