# Supplementary material for: Model design choices impact biological insight: Unpacking the broad landscape of spatial-temporal model development decisions
Source: PLoS Comput Biol. 2024 Mar 8;20(3):e1011917. doi: 10.1371/journal.pcbi.1011917 (PMC10954156; doi:10.1371/journal.pcbi.1011917)

S4 Fig. Cell variability emergent behavior. *Related to Fig 4.* (**A**) Time course of emergent metrics by volume. (**B**) Time course of emergent metrics by age.

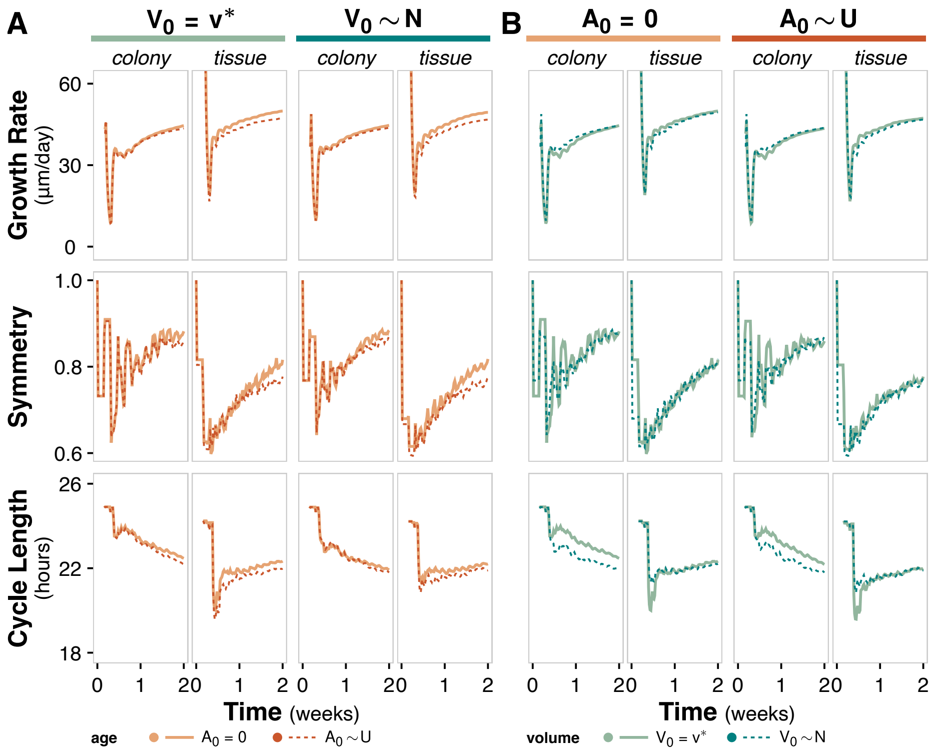

Supplement: S4 Fig — (PDF) [file pcbi.1011917.s004.pdf]
