# Supplementary material for: Model design choices impact biological insight: Unpacking the broad landscape of spatial-temporal model development decisions
Source: PLoS Comput Biol. 2024 Mar 8;20(3):e1011917. doi: 10.1371/journal.pcbi.1011917 (PMC10954156; doi:10.1371/journal.pcbi.1011917)

S6 Fig. **Nutrient dynamics emergent behavior**. *Related to Fig 6.* (**A**) Time course of emergent metrics by volume. (**B**) Time course of emergent metrics by age.

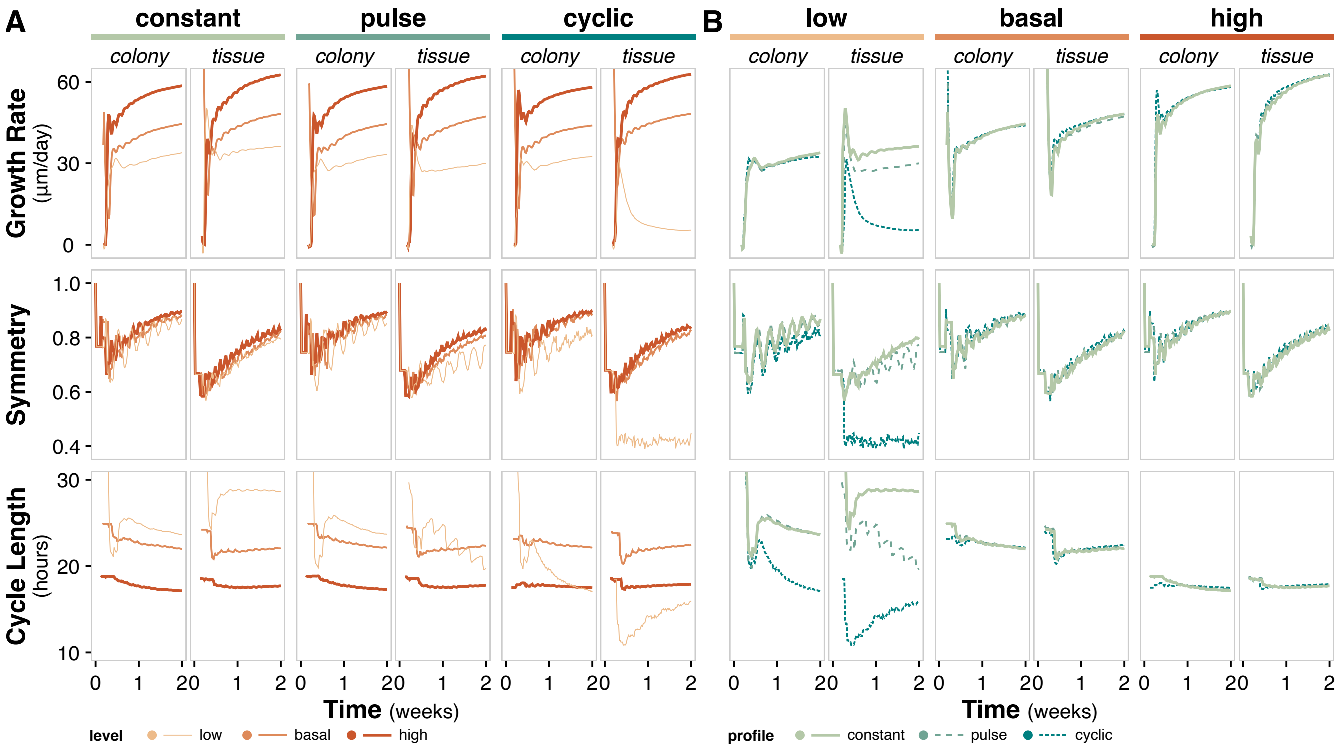

Supplement: S6 Fig — (PDF) [file pcbi.1011917.s006.pdf]
