# Supplementary material for: Model design choices impact biological insight: Unpacking the broad landscape of spatial-temporal model development decisions
Source: PLoS Comput Biol. 2024 Mar 8;20(3):e1011917. doi: 10.1371/journal.pcbi.1011917 (PMC10954156; doi:10.1371/journal.pcbi.1011917)

S7 Fig. **Model framework and geometry**. (**A**) Diagram of added and modified model classes. Numbered circles indicate which classes where added or modified for specific simulation conditions. (**B**) Diagram of rectangular and hexagonal geometry for the cell grid and the environment lattices.

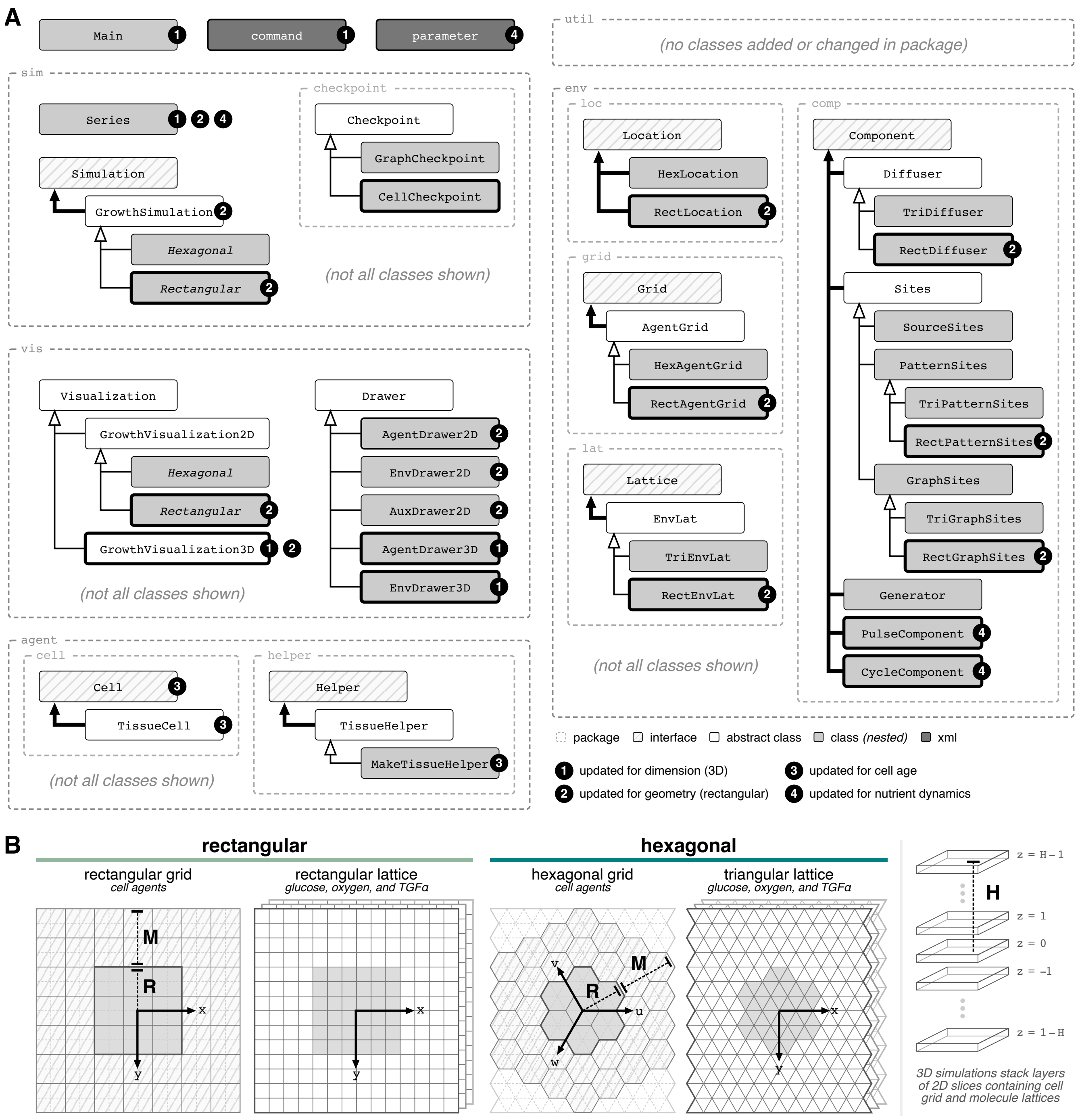

Supplement: S7 Fig — (PDF) [file pcbi.1011917.s007.pdf]
