# Supplementary material for: Model design choices impact biological insight: Unpacking the broad landscape of spatial-temporal model development decisions
Source: PLoS Comput Biol. 2024 Mar 8;20(3):e1011917. doi: 10.1371/journal.pcbi.1011917 (PMC10954156; doi:10.1371/journal.pcbi.1011917)
Supplement: S1 Table — (PDF) [file pcbi.1011917.s008.pdf]

S1 Table. Input options used to run simulations. For clarity, wrapping <set> tags and <series> name attributes are not shown. Lines highlighted in gray are only included in simulations for the indicated condition (left) and/or context (right). For each set, simulations were run for every combination of conditions. All simulations are run with two contexts: *colony* (only cancer cells) and *tissue* (cancer and healthy tissue cells). Cancer cell agents are added after a delay of 1440 ticks (1 day).

(**A**) System representation

	<series start="0" end="50" days="15">	
2D	<simulation type="growth" radius="**34**" margin="6" height="**1**">	
3D	<simulation type="growth" radius="**26**" margin="6" height="**51**">	
	<profilers>	
	<profiler type="growth" interval="720" suffix="" />	
	</profilers>	
	</simulation>	
	<agents initialization="0">	*colony*
	<agents initialization="FULL">	*tissue*
	<populations>	
	<population type="C" fraction="0.0">	
	<variables>	
	<variable id="max_height" scale="1.5" />	
	<variable id="meta_pref" scale="1.5" />	
	<variable id="migra_threshold" scale="0.5" />	
	</variables>	
	</population>	
	<population type="H" fraction="1.0" />	*tissue*
	</populations>	
	<helpers>	
	<helper type="insert" delay="1440" populations="0" bounds="0.05" />	
	</helpers>	
	</agents>	
rectangular	<environment coordinate="**rect**">	
hexagonal	<environment coordinate="**hex**">	
	<components>	
	<component type="sites" class="source" />	
	</components>	
	</environment>	
	</series>	

(**B**) Cell variability

	<series start="0" end="50" days="15">	
	<simulation type="growth">	
	<profilers>	
	<profiler type="growth" interval="180" suffix="" />	
	</profilers>	
	</simulation>	
	<agents initialization="0">	*colony*
	<agents initialization="FULL">	*tissue*
	<populations>	
	<population type="C" fraction="0.0">	
	<variables>	
	<variable id="max_height" scale="1.5" />	
	<variable id="meta_pref" scale="1.5" />	
	<variable id="migra_threshold" scale="0.5" />	
$V_0 = v^*$	<variable id="cell_vol_range" scale="**0.0**" />	
$V_0 \sim N$	<variable id="cell_vol_range" scale="**1.0**" />	
$A_0 = 0$	<variable id="cell_age_max" scale="**0.0**" />	
$A_0 \sim U$	<variable id="cell_age_max" scale="**1.0**" />	
	</variables>	
	</population>	
	<population type="H" fraction="1.0">	*tissue*
	<variables>	*tissue*
$V_0 = v^*$	<variable id="cell_vol_range" scale="**0.0**" />	*tissue*
$V_0 \sim N$	<variable id="cell_vol_range" scale="**1.0**" />	*tissue*
$A_0 = 0$	<variable id="cell_age_max" scale="**0.0**" />	*tissue*
$A_0 \sim U$	<variable id="cell_age_max" scale="**1.0**" />	*tissue*
	</variables>	*tissue*
	</population>	*tissue*
	</populations>	
	<helpers>	
	<helper type="insert" delay="1440" populations="0" bounds="0.05" />	
	</helpers>	
	</agents>	
	<environment coordinate="hex">	
	<components>	
	<component type="sites" class="source" />	
	</components>	
	</environment>	
	</series>	

(C) Nutrient dynamics

```
         <series start="0" end="50" days="15">
           <simulation type="growth">
             <profilers>
               <profiler type="growth" interval="180" suffix="" />
             </profilers>
           </simulation>
           <agents initialization="0">                                                   colony
           <agents initialization="FULL">                                                tissue
             <populations>
               <population type="C" fraction="0.0">
                 <variables>
                   <variable id="max_height" scale="1.5" />
                   <variable id="meta_pref" scale="1.5" />
                   <variable id="migra_threshold" scale="0.5" />
                 </variables>
               </population>
               <population type="H" fraction="1.0" />                                     tissue
             </populations>
             <helpers>
               <helper type="insert" delay="1440" populations="0" bounds="0.05" />
             </helpers>
           </agents>
           <environment coordinate="hex">
             <globals>
low            <global id="concentration_glucose" scale="0.5" />
basal          <global id="concentration_glucose" scale="1.0" />
high           <global id="concentration_glucose" scale="2.0" />
             </globals>
             <components>
               <component type="sites" class="source" />
pulse          <component type="pulse" />
cyclic         <component type="cycle" />
             </components>
           </environment>
         </series>
```
