# Supplementary material for: Model design choices impact biological insight: Unpacking the broad landscape of spatial-temporal model development decisions
Source: PLoS Comput Biol. 2024 Mar 8;20(3):e1011917. doi: 10.1371/journal.pcbi.1011917 (PMC10954156; doi:10.1371/journal.pcbi.1011917)
Supplement: S2 Table — (PDF) [file pcbi.1011917.s009.pdf]

S2 Table. **CPU time for simulations.** Simulations are run on nodes with Intel® Haswell (24x 2.50 GHz), Intel® Xeon (28x 2.40 GHz), and Intel® Xeon Gold (28x 2.60 GHz) processors and 128 GB of RAM per node.

(**A**) System representation

colony context

**Factor**	**Level**	**CPU Time** (minutes)			**N**
geometry	rectangular	381.8	±	379.4	100
geometry	hexagonal	560.5	±	556.6	100
dimension	2D	9.6	±	2.1	100
dimension	3DC • 3D	932.7	±	198.6	100

tissue context

**Factor**	**Level**	**CPU Time** (minutes)			**N**
geometry	rectangular	1643.5	±	1640.6	100
geometry	hexagonal	1398.2	±	1395.2	100
dimension	2D	16.8	±	2.5	100
dimension	3DC • 3D	3024.9	±	314.5	100

(**B**) Cell variability

colony context

**Factor**	**Level**	**CPU Time** (minutes)			**N**
volume	$V_0 = v^*$	10.1	±	1.3	100
volume	$V_0 \sim N$	10.0	±	1.1	100
age	$A_0 = 0$	10.1	±	1.2	100
age	$A_0 \sim U$	10.0	±	1.2	100

tissue context

**Factor**	**Level**	**CPU Time** (minutes)			**N**
volume	$V_0 = v^*$	18.4	±	1.6	100
volume	$V_0 \sim N$	17.8	±	2.0	100
age	$A_0 = 0$	18.0	±	1.8	100
age	$A_0 \sim U$	18.2	±	1.9	100

(**C**) Nutrient dynamics

colony context

**Factor**	**Level**	**CPU Time** (minutes)			**N**
profile	constant	10.5	±	0.8	150
profile	pulse	10.5	±	1.2	150
profile	cyclic	10.4	±	1.2	150
level	low	10.1	±	1.1	150
level	basal	10.0	±	0.7	150
level	high	11.3	±	0.9	150

tissue context

**Factor**	**Level**	**CPU Time** (minutes)			**N**
profile	constant	16.9	±	1.7	150
profile	pulse	19.1	±	2.7	150
profile	cyclic	17.6	±	2.4	150
level	low	17.7	±	2.9	150
level	basal	17.3	±	2.3	150
level	high	18.5	±	2.1	150
