# Supplementary material for: Model design choices impact biological insight: Unpacking the broad landscape of spatial-temporal model development decisions
Source: PLoS Comput Biol. 2024 Mar 8;20(3):e1011917. doi: 10.1371/journal.pcbi.1011917 (PMC10954156; doi:10.1371/journal.pcbi.1011917)
Supplement: S3 Table — (PDF) [file pcbi.1011917.s010.pdf]

S3 Table. Cell and effect means for system representation emergent metrics.

(**A**) Growth Rate (μm/day)

colony context

MEANS AND STANDARD DEVIATIONS

Geometry	Dimension	Mean	SD	N
rectangular	2D	38.531	1.026	50
rectangular	3DC	42.547	1.039	50
rectangular	3D	42.190	0.577	50
hexagonal	2D	44.288	1.104	50
hexagonal	3DC	45.313	1.141	50
hexagonal	3D	44.772	0.840	50

EFFECT MEANS AND STANDARD DEVIATIONS

Factor	Level	Mean	SD	N
geometry	rectangular	41.089	2.031	150
geometry	hexagonal	44.791	1.112	150
dimension	2D	41.410	3.081	100
dimension	3DC	43.930	1.764	100
dimension	3D	43.481	1.482	100

tissue context

MEANS AND STANDARD DEVIATIONS

Geometry	Dimension	Mean	SD	N
rectangular	2D	39.200	1.484	50
rectangular	3DC	44.581	1.124	50
rectangular	3D	43.800	0.705	50
hexagonal	2D	48.474	1.801	50
hexagonal	3DC	48.844	1.200	50
hexagonal	3D	48.481	0.830	50

EFFECT MEANS AND STANDARD DEVIATIONS

Factor	Level	Mean	SD	N
geometry	rectangular	42.527	2.641	150
geometry	hexagonal	48.600	1.340	150
dimension	2D	43.837	4.941	100
dimension	3DC	46.712	2.435	100
dimension	3D	46.140	2.474	100

(**B**) Symmetry

colony context

MEANS AND STANDARD DEVIATIONS

Geometry	Dimension	Mean	SD	N
rectangular	2D	0.884	0.030	50
rectangular	3DC	0.884	0.023	50
rectangular	3D	0.809	0.017	50
hexagonal	2D	0.886	0.036	50
hexagonal	3DC	0.866	0.025	50
hexagonal	3D	0.766	0.021	50

EFFECT MEANS AND STANDARD DEVIATIONS

Factor	Level	Mean	SD	N
geometry	rectangular	0.859	0.043	150
geometry	hexagonal	0.839	0.060	150
dimension	2D	0.885	0.033	100
dimension	3DC	0.875	0.026	100
dimension	3D	0.788	0.029	100

tissue context

MEANS AND STANDARD DEVIATIONS

Geometry	Dimension	Mean	SD	N
rectangular	2D	0.829	0.042	50
rectangular	3DC	0.863	0.026	50
rectangular	3D	0.777	0.019	50
hexagonal	2D	0.813	0.030	50
hexagonal	3DC	0.835	0.023	50
hexagonal	3D	0.727	0.017	50

EFFECT MEANS AND STANDARD DEVIATIONS

Factor	Level	Mean	SD	N
geometry	rectangular	0.823	0.047	150
geometry	hexagonal	0.791	0.053	150
dimension	2D	0.821	0.037	100
dimension	3DC	0.849	0.028	100
dimension	3D	0.752	0.031	100

(**C**) Cycle Length (hours)

colony context

MEANS AND STANDARD DEVIATIONS

Geometry	Dimension	Mean	SD	N
rectangular	2D	22.223	0.365	50
rectangular	3DC	21.924	0.259	50
rectangular	3D	21.806	0.114	50
hexagonal	2D	21.941	0.559	50
hexagonal	3DC	21.686	0.381	50
hexagonal	3D	21.629	0.210	50

EFFECT MEANS AND STANDARD DEVIATIONS

Factor	Level	Mean	SD	N
geometry	rectangular	21.984	0.318	150
geometry	hexagonal	21.752	0.428	150
dimension	2D	22.082	0.491	100
dimension	3DC	21.805	0.345	100
dimension	3D	21.717	0.190	100

tissue context

MEANS AND STANDARD DEVIATIONS

Geometry	Dimension	Mean	SD	N
rectangular	2D	22.451	0.428	50
rectangular	3DC	21.798	0.217	50
rectangular	3D	21.802	0.106	50
hexagonal	2D	22.176	0.552	50
hexagonal	3DC	21.711	0.336	50
hexagonal	3D	21.795	0.127	50

EFFECT MEANS AND STANDARD DEVIATIONS

Factor	Level	Mean	SD	N
geometry	rectangular	22.017	0.417	150
geometry	hexagonal	21.894	0.429	150
dimension	2D	22.314	0.511	100
dimension	3DC	21.755	0.285	100
dimension	3D	21.799	0.117	100
