# Supplementary material for: Model design choices impact biological insight: Unpacking the broad landscape of spatial-temporal model development decisions
Source: PLoS Comput Biol. 2024 Mar 8;20(3):e1011917. doi: 10.1371/journal.pcbi.1011917 (PMC10954156; doi:10.1371/journal.pcbi.1011917)
Supplement: S4 Table — (PDF) [file pcbi.1011917.s011.pdf]

S4 Table. ANOVA for system representation emergent metrics.

(**A**) Growth Rate (μm/day)

colony context

TWO-WAY ANOVA WITH INTERACTION

	SS	DF	MSE	F	P
**Geometry**	1027.6	1	1027.6	1083.1	**0.000**
**Dimension**	361.4	2	180.7	190.4	**0.000**
**Interaction**	158.8	2	79.4	83.7	**0.000**
Residual	278.9	294	0.9	–	–
Total	1826.7	299	–	–	–

SIMPLE MAIN EFFECTS TESTING : GEOMETRY

Dimension	SS	DF	MSE	F	P
2D	828.5	1	828.5	873.2	**0.000**
3DC	191.3	1	191.3	201.6	**0.000**
3D	166.7	1	166.7	175.7	**0.000**

SIMPLE MAIN EFFECTS TESTING : DIMENSION

Geometry	SS	DF	MSE	F	P
rectangular	493.9	2	247.0	260.3	**0.000**
hexagonal	26.3	2	13.1	13.8	**0.000**

TUKEY MULTIPLE COMPARISON : DIMENSION

Geometry	A	B	MD	P
rectangular	2D	3DC	4.015	**0.001**
rectangular	2D	3D	3.659	**0.001**
rectangular	3D	3DC	0.357	0.124
hexagonal	2D	3DC	1.024	**0.001**
hexagonal	2D	3D	0.484	0.055
hexagonal	3D	3DC	0.541	0.027

tissue context

TWO-WAY ANOVA WITH INTERACTION

	SS	DF	MSE	F	P
**Geometry**	2766.4	1	2766.4	1778.3	**0.000**
**Dimension**	463.3	2	231.7	148.9	**0.000**
**Interaction**	386.5	2	193.3	124.2	**0.000**
Residual	457.4	294	1.6	–	–
Total	4073.6	299	–	–	–

SIMPLE MAIN EFFECTS TESTING : GEOMETRY

Dimension	SS	DF	MSE	F	P
2D	2150.5	1	2150.5	1382.4	**0.000**
3DC	454.4	1	454.4	292.1	**0.000**
3D	547.9	1	547.9	352.2	**0.000**

SIMPLE MAIN EFFECTS TESTING : DIMENSION

Geometry	SS	DF	MSE	F	P
rectangular	845.4	2	422.7	271.7	**0.000**
hexagonal	4.5	2	2.2	1.4	0.239

TUKEY MULTIPLE COMPARISON : DIMENSION

Geometry	A	B	MD	P
rectangular	2D	3DC	5.381	**0.001**
rectangular	2D	3D	4.600	**0.001**
rectangular	3D	3DC	0.781	**0.003**
hexagonal	2D	3DC	0.370	0.354
hexagonal	2D	3D	0.007	0.900
hexagonal	3D	3DC	0.363	0.368

(**B**) Symmetry

colony context

TWO-WAY ANOVA WITH INTERACTION

	SS	DF	MSE	F	P
**Geometry**	0.0	1	0.0	45.4	**0.000**
**Dimension**	0.6	2	0.3	423.9	**0.000**
**Interaction**	0.0	2	0.0	18.9	**0.000**
Residual	0.2	294	0.0	–	–
Total	0.8	299	–	–	–

SIMPLE MAIN EFFECTS TESTING : GEOMETRY

Dimension	SS	DF	MSE	F	P
2D	0.0	1	0.0	0.1	0.814
3DC	0.0	1	0.0	12.0	**0.001**
3D	0.0	1	0.0	71.2	**0.000**

SIMPLE MAIN EFFECTS TESTING : DIMENSION

Geometry	SS	DF	MSE	F	P
rectangular	0.2	2	0.1	137.0	**0.000**
hexagonal	0.4	2	0.2	305.8	**0.000**

TUKEY MULTIPLE COMPARISON : DIMENSION

Geometry	A	B	MD	P
rectangular	2D	3DC	-0.001	0.900
rectangular	2D	3D	-0.075	**0.001**
rectangular	3D	3DC	0.074	**0.001**
hexagonal	2D	3DC	-0.020	**0.001**
hexagonal	2D	3D	-0.120	**0.001**
hexagonal	3D	3DC	0.100	**0.001**

tissue context

TWO-WAY ANOVA WITH INTERACTION

	SS	DF	MSE	F	P
**Geometry**	0.1	1	0.1	100.5	**0.000**
**Dimension**	0.5	2	0.2	332.4	**0.000**
**Interaction**	0.0	2	0.0	9.7	**0.000**
Residual	0.2	294	0.0	–	–
Total	0.8	299	–	–	–

SIMPLE MAIN EFFECTS TESTING : GEOMETRY

Dimension	SS	DF	MSE	F	P
2D	0.0	1	0.0	9.4	**0.002**
3DC	0.0	1	0.0	26.0	**0.000**
3D	0.1	1	0.1	84.6	**0.000**

SIMPLE MAIN EFFECTS TESTING : DIMENSION

Geometry	SS	DF	MSE	F	P
rectangular	0.2	2	0.1	124.5	**0.000**
hexagonal	0.3	2	0.2	217.7	**0.000**

TUKEY MULTIPLE COMPARISON : DIMENSION

Geometry	A	B	MD	P
rectangular	2D	3DC	0.033	**0.001**
rectangular	2D	3D	-0.052	**0.001**
rectangular	3D	3DC	0.086	**0.001**
hexagonal	2D	3DC	0.022	**0.001**
hexagonal	2D	3D	-0.086	**0.001**
hexagonal	3D	3DC	0.108	**0.001**

(**C**) Cycle Length (hours)

colony context

TWO-WAY ANOVA WITH INTERACTION

	SS	DF	MSE	F	P
**Geometry**	4.1	1	4.1	34.0	**0.000**
**Dimension**	7.2	2	3.6	30.4	**0.000**
**Interaction**	0.1	2	0.1	0.6	0.555
Residual	35.0	294	0.1	–	–
Total	46.4	299	–	–	–

TWO-WAY ANOVA WITHOUT INTERACTION

	SS	DF	MSE	F	P
**Geometry**	4.1	1	4.1	34.1	**0.000**
**Dimension**	7.2	2	3.6	30.5	**0.000**
Residual	35.1	296	0.1	–	–
Total	46.4	299	–	–	–

TUKEY MULTIPLE COMPARISON : DIMENSION

A	B	MD	P
2D	3DC	-0.277	**0.001**
2D	3D	-0.365	**0.001**
3D	3DC	0.087	0.206

tissue context

TWO-WAY ANOVA WITH INTERACTION

	SS	DF	MSE	F	P
**Geometry**	1.1	1	1.1	10.2	**0.002**
**Dimension**	19.3	2	9.7	85.8	**0.000**
**Interaction**	0.9	2	0.5	4.2	**0.016**
Residual	33.1	294	0.1	–	–
Total	54.5	299	–	–	–

SIMPLE MAIN EFFECTS TESTING : GEOMETRY

Dimension	SS	DF	MSE	F	P
2D	1.9	1	1.9	16.8	**0.000**
3DC	0.2	1	0.2	1.7	0.193
3D	0.0	1	0.0	0.0	0.909

SIMPLE MAIN EFFECTS TESTING : DIMENSION

Geometry	SS	DF	MSE	F	P
rectangular	14.1	2	7.1	62.7	**0.000**
hexagonal	6.1	2	3.1	27.3	**0.000**

TUKEY MULTIPLE COMPARISON : DIMENSION

Geometry	A	B	MD	P
rectangular	2D	3DC	-0.653	**0.001**
rectangular	2D	3D	-0.649	**0.001**
rectangular	3D	3DC	-0.004	0.900
hexagonal	2D	3DC	-0.465	**0.001**
hexagonal	2D	3D	-0.381	**0.001**
hexagonal	3D	3DC	-0.084	0.514
