# Supplementary material for: Model design choices impact biological insight: Unpacking the broad landscape of spatial-temporal model development decisions
Source: PLoS Comput Biol. 2024 Mar 8;20(3):e1011917. doi: 10.1371/journal.pcbi.1011917 (PMC10954156; doi:10.1371/journal.pcbi.1011917)
Supplement: S5 Table — (PDF) [file pcbi.1011917.s012.pdf]

S5 Table. Cell and effect means for cell variability emergent metrics.

(**A**) Growth Rate (μm/day)

colony context

MEANS AND STANDARD DEVIATIONS

Volume	Age	Mean	SD	N
$V_0 = v^*$	$A_0 = 0$	44.554	1.021	50
$V_0 = v^*$	$A_0 \sim U$	43.516	1.705	50
$V_0 \sim N$	$A_0 = 0$	44.661	0.981	50
$V_0 \sim N$	$A_0 \sim U$	43.749	1.780	50

EFFECT MEANS AND STANDARD DEVIATIONS

Factor	Level	Mean	SD	N
volume	$V_0 = v^*$	44.035	1.493	100
volume	$V_0 \sim N$	44.205	1.502	100
age	$A_0 = 0$	44.608	0.998	100
age	$A_0 \sim U$	43.633	1.738	100

tissue context

MEANS AND STANDARD DEVIATIONS

Volume	Age	Mean	SD	N
$V_0 = v^*$	$A_0 = 0$	49.925	1.441	50
$V_0 = v^*$	$A_0 \sim U$	47.270	3.064	50
$V_0 \sim N$	$A_0 = 0$	49.426	1.704	50
$V_0 \sim N$	$A_0 \sim U$	46.741	2.908	50

EFFECT MEANS AND STANDARD DEVIATIONS

Factor	Level	Mean	SD	N
volume	$V_0 = v^*$	48.597	2.730	100
volume	$V_0 \sim N$	48.084	2.728	100
age	$A_0 = 0$	49.675	1.590	100
age	$A_0 \sim U$	47.006	2.983	100

(**B**) Symmetry

colony context

MEANS AND STANDARD DEVIATIONS

Volume	Age	Mean	SD	N
$V_0 = v^*$	$A_0 = 0$	0.882	0.026	50
$V_0 = v^*$	$A_0 \sim U$	0.859	0.054	50
$V_0 \sim N$	$A_0 = 0$	0.884	0.024	50
$V_0 \sim N$	$A_0 \sim U$	0.866	0.042	50

EFFECT MEANS AND STANDARD DEVIATIONS

Factor	Level	Mean	SD	N
volume	$V_0 = v^*$	0.871	0.044	100
volume	$V_0 \sim N$	0.875	0.035	100
age	$A_0 = 0$	0.883	0.025	100
age	$A_0 \sim U$	0.862	0.048	100

tissue context

MEANS AND STANDARD DEVIATIONS

Volume	Age	Mean	SD	N
$V_0 = v^*$	$A_0 = 0$	0.816	0.029	50
$V_0 = v^*$	$A_0 \sim U$	0.777	0.064	50
$V_0 \sim N$	$A_0 = 0$	0.818	0.034	50
$V_0 \sim N$	$A_0 \sim U$	0.774	0.080	50

EFFECT MEANS AND STANDARD DEVIATIONS

Factor	Level	Mean	SD	N
volume	$V_0 = v^*$	0.796	0.053	100
volume	$V_0 \sim N$	0.796	0.065	100
age	$A_0 = 0$	0.817	0.031	100
age	$A_0 \sim U$	0.775	0.072	100

(**C**) Cycle Length (hours)

colony context

MEANS AND STANDARD DEVIATIONS

Volume	Age	Mean	SD	N
$V_0 = v^*$	$A_0 = 0$	22.469	0.456	50
$V_0 = v^*$	$A_0 \sim U$	22.181	0.629	50
$V_0 \sim N$	$A_0 = 0$	21.922	0.493	50
$V_0 \sim N$	$A_0 \sim U$	21.799	0.598	50

EFFECT MEANS AND STANDARD DEVIATIONS

Factor	Level	Mean	SD	N
volume	$V_0 = v^*$	22.325	0.566	100
volume	$V_0 \sim N$	21.861	0.549	100
age	$A_0 = 0$	22.195	0.547	100
age	$A_0 \sim U$	21.990	0.640	100

tissue context

MEANS AND STANDARD DEVIATIONS

Volume	Age	Mean	SD	N
$V_0 = v^*$	$A_0 = 0$	22.335	0.367	50
$V_0 = v^*$	$A_0 \sim U$	21.981	0.434	50
$V_0 \sim N$	$A_0 = 0$	22.206	0.527	50
$V_0 \sim N$	$A_0 \sim U$	21.905	0.619	50

EFFECT MEANS AND STANDARD DEVIATIONS

Factor	Level	Mean	SD	N
volume	$V_0 = v^*$	22.158	0.438	100
volume	$V_0 \sim N$	22.055	0.592	100
age	$A_0 = 0$	22.271	0.457	100
age	$A_0 \sim U$	21.943	0.533	100
