# Supplementary material for: Model design choices impact biological insight: Unpacking the broad landscape of spatial-temporal model development decisions
Source: PLoS Comput Biol. 2024 Mar 8;20(3):e1011917. doi: 10.1371/journal.pcbi.1011917 (PMC10954156; doi:10.1371/journal.pcbi.1011917)
Supplement: S6 Table — (PDF) [file pcbi.1011917.s013.pdf]

S6 Table. ANOVA for cell variability emergent metrics.

(**A**) Growth Rate (μm/day)

colony context

TWO-WAY ANOVA WITH INTERACTION

	SS	DF	MSE	F	P
**Volume**	1.4	1	1.4	0.7	0.399
**Age**	47.5	1	47.5	23.5	**0.000**
**Interaction**	0.2	1	0.2	0.1	0.755
Residual	396.1	196	2.0	–	–
Total	445.3	199	–	–	–

TWO-WAY ANOVA WITHOUT INTERACTION

	SS	DF	MSE	F	P
**Volume**	1.4	1	1.4	0.7	0.398
**Age**	47.5	1	47.5	23.6	**0.000**
Residual	396.3	197	2.0	–	–
Total	445.3	199	–	–	–

tissue context

TWO-WAY ANOVA WITH INTERACTION

	SS	DF	MSE	F	P
**Volume**	13.2	1	13.2	2.3	0.130
**Age**	356.3	1	356.3	62.5	**0.000**
**Interaction**	0.0	1	0.0	0.0	0.965
Residual	1118.3	196	5.7	–	–
Total	1487.8	199	–	–	–

TWO-WAY ANOVA WITHOUT INTERACTION

	SS	DF	MSE	F	P
**Volume**	13.2	1	13.2	2.3	0.129
**Age**	356.3	1	356.3	62.8	**0.000**
Residual	1118.3	197	5.7	–	–
Total	1487.8	199	–	–	–

(**B**) Symmetry

colony context

TWO-WAY ANOVA WITH INTERACTION

	SS	DF	MSE	F	P
**Volume**	0.0	1	0.0	0.6	0.437
**Age**	0.0	1	0.0	14.3	**0.000**
**Interaction**	0.0	1	0.0	0.2	0.694
Residual	0.3	196	0.0	–	–
Total	0.3	199	–	–	–

TWO-WAY ANOVA WITHOUT INTERACTION

	SS	DF	MSE	F	P
**Volume**	0.0	1	0.0	0.6	0.436
**Age**	0.0	1	0.0	14.4	**0.000**
Residual	0.3	197	0.0	–	–
Total	0.3	199	–	–	–

tissue context

TWO-WAY ANOVA WITH INTERACTION

	SS	DF	MSE	F	P
**Volume**	0.0	1	0.0	0.0	0.937
**Age**	0.1	1	0.1	27.6	**0.000**
**Interaction**	0.0	1	0.0	0.1	0.751
Residual	0.6	196	0.0	–	–
Total	0.7	199	–	–	–

TWO-WAY ANOVA WITHOUT INTERACTION

	SS	DF	MSE	F	P
**Volume**	0.0	1	0.0	0.0	0.937
**Age**	0.1	1	0.1	27.7	**0.000**
Residual	0.6	197	0.0	–	–
Total	0.7	199	–	–	–

(**C**) Cycle Length (hours)

colony context

TWO-WAY ANOVA WITH INTERACTION

	SS	DF	MSE	F	P
**Volume**	10.8	1	10.8	35.8	**0.000**
**Age**	2.1	1	2.1	7.0	**0.009**
**Interaction**	0.3	1	0.3	1.1	0.286
Residual	59.0	196	0.3	–	–
Total	72.3	199	–	–	–

TWO-WAY ANOVA WITHOUT INTERACTION

	SS	DF	MSE	F	P
**Volume**	10.8	1	10.8	35.8	**0.000**
**Age**	2.1	1	2.1	7.0	**0.009**
Residual	59.4	197	0.3	–	–
Total	72.3	199	–	–	–

tissue context

TWO-WAY ANOVA WITH INTERACTION

	SS	DF	MSE	F	P
**Volume**	0.5	1	0.5	2.1	0.145
**Age**	5.4	1	5.4	21.8	**0.000**
**Interaction**	0.0	1	0.0	0.1	0.710
Residual	48.2	196	0.2	–	–
Total	54.2	199	–	–	–

TWO-WAY ANOVA WITHOUT INTERACTION

	SS	DF	MSE	F	P
**Volume**	0.5	1	0.5	2.2	0.144
**Age**	5.4	1	5.4	21.9	**0.000**
Residual	48.3	197	0.2	–	–
Total	54.2	199	–	–	–
