# Supplementary material for: Model design choices impact biological insight: Unpacking the broad landscape of spatial-temporal model development decisions
Source: PLoS Comput Biol. 2024 Mar 8;20(3):e1011917. doi: 10.1371/journal.pcbi.1011917 (PMC10954156; doi:10.1371/journal.pcbi.1011917)
Supplement: S7 Table — (PDF) [file pcbi.1011917.s014.pdf]

S7 Table. Cell and effect means for nutrient dynamics emergent metrics.

(**A**) Growth Rate (μm/day)

colony context

MEANS AND STANDARD DEVIATIONS

Profile	Level	Mean	SD	N
constant	low	33.874	0.609	50
constant	basal	44.535	0.885	50
constant	high	58.553	1.176	50
pulse	low	33.364	0.902	50
pulse	basal	44.499	1.024	50
pulse	high	58.355	1.068	50
cyclic	low	32.487	1.167	50
cyclic	basal	43.943	0.961	50
cyclic	high	58.025	1.253	50

EFFECT MEANS AND STANDARD DEVIATIONS

Factor	Level	Mean	SD	N
profile	constant	45.654	10.181	150
profile	pulse	45.406	10.305	150
profile	cyclic	44.818	10.540	150
level	low	33.242	1.081	150
level	basal	44.326	0.990	150
level	high	58.311	1.181	150

tissue context

MEANS AND STANDARD DEVIATIONS

Profile	Level	Mean	SD	N
constant	low	36.176	1.578	50
constant	basal	48.126	1.823	50
constant	high	62.645	1.714	50
pulse	low	29.964	2.620	50
pulse	basal	47.221	1.781	50
pulse	high	62.132	1.710	50
cyclic	low	5.366	3.373	50
cyclic	basal	48.201	1.701	50
cyclic	high	62.828	1.966	50

EFFECT MEANS AND STANDARD DEVIATIONS

Factor	Level	Mean	SD	N
profile	constant	48.982	10.991	150
profile	pulse	46.439	13.349	150
profile	cyclic	38.798	24.586	150
level	low	23.836	13.601	150
level	basal	47.849	1.813	150
level	high	62.535	1.812	150

(**B**) Symmetry

colony context

MEANS AND STANDARD DEVIATIONS

Profile	Level	Mean	SD	N
constant	low	0.868	0.030	50
constant	basal	0.883	0.026	50
constant	high	0.897	0.022	50
pulse	low	0.850	0.030	50
pulse	basal	0.883	0.028	50
pulse	high	0.896	0.021	50
cyclic	low	0.811	0.034	50
cyclic	basal	0.888	0.026	50
cyclic	high	0.900	0.025	50

EFFECT MEANS AND STANDARD DEVIATIONS

Factor	Level	Mean	SD	N
profile	constant	0.883	0.029	150
profile	pulse	0.877	0.033	150
profile	cyclic	0.866	0.049	150
level	low	0.843	0.040	150
level	basal	0.885	0.027	150
level	high	0.898	0.023	150

tissue context

MEANS AND STANDARD DEVIATIONS

Profile	Level	Mean	SD	N
constant	low	0.794	0.033	50
constant	basal	0.825	0.033	50
constant	high	0.825	0.021	50
pulse	low	0.772	0.050	50
pulse	basal	0.806	0.033	50
pulse	high	0.825	0.027	50
cyclic	low	0.448	0.116	50
cyclic	basal	0.823	0.031	50
cyclic	high	0.840	0.026	50

EFFECT MEANS AND STANDARD DEVIATIONS

Factor	Level	Mean	SD	N
profile	constant	0.815	0.032	150
profile	pulse	0.801	0.044	150
profile	cyclic	0.704	0.195	150
level	low	0.671	0.176	150
level	basal	0.818	0.033	150
level	high	0.830	0.026	150

(**C**) Cycle Length (hours)

colony context

MEANS AND STANDARD DEVIATIONS

Profile	Level	Mean	SD	N
constant	low	23.653	1.181	50
constant	basal	21.993	0.530	50
constant	high	17.143	0.269	50
pulse	low	23.687	1.284	50
pulse	basal	22.126	0.567	50
pulse	high	17.291	0.305	50
cyclic	low	17.018	0.798	50
cyclic	basal	22.125	0.631	50
cyclic	high	17.484	0.273	50

EFFECT MEANS AND STANDARD DEVIATIONS

Factor	Level	Mean	SD	N
profile	constant	20.929	2.873	150
profile	pulse	21.035	2.854	150
profile	cyclic	18.876	2.391	150
level	low	21.453	3.333	150
level	basal	22.081	0.577	150
level	high	17.306	0.314	150

tissue context

MEANS AND STANDARD DEVIATIONS

Profile	Level	Mean	SD	N
constant	low	28.673	0.636	50
constant	basal	22.092	0.477	50
constant	high	17.707	0.245	50
pulse	low	19.708	1.341	50
pulse	basal	22.349	0.521	50
pulse	high	17.787	0.262	50
cyclic	low	15.972	4.020	47
cyclic	basal	22.454	0.502	50
cyclic	high	17.889	0.177	50

EFFECT MEANS AND STANDARD DEVIATIONS

Factor	Level	Mean	SD	N
profile	constant	22.824	4.547	150
profile	pulse	19.948	2.055	150
profile	cyclic	18.828	3.552	147
level	low	21.563	5.864	147
level	basal	22.298	0.520	150
level	high	17.794	0.241	150
