# Supplementary material for: Model design choices impact biological insight: Unpacking the broad landscape of spatial-temporal model development decisions
Source: PLoS Comput Biol. 2024 Mar 8;20(3):e1011917. doi: 10.1371/journal.pcbi.1011917 (PMC10954156; doi:10.1371/journal.pcbi.1011917)
Supplement: S8 Table — (PDF) [file pcbi.1011917.s015.pdf]

S8 Table. ANOVA for nutrient dynamics emergent metrics.

(**A**) Growth Rate (μm/day)

colony context

TWO-WAY ANOVA WITH INTERACTION

	SS	DF	MSE	F	P
**Profile**	55.3	2	27.6	26.5	**0.000**
**Level**	47347.0	2	23673.5	22680.3	**0.000**
**Interaction**	12.1	4	3.0	2.9	**0.022**
Residual	460.3	441	1.0	–	–
Total	47874.7	449	–	–	–

SIMPLE MAIN EFFECTS TESTING : PROFILE

Level	SS	DF	MSE	F	P
low	49.2	2	24.6	23.6	**0.000**
basal	11.0	2	5.5	5.3	**0.005**
high	7.1	2	3.6	3.4	0.034

SIMPLE MAIN EFFECTS TESTING : LEVEL

Profile	SS	DF	MSE	F	P
constant	15320.5	2	7660.3	7338.9	**0.000**
pulse	15675.7	2	7837.8	7509.0	**0.000**
cyclic	16362.9	2	8181.5	7838.2	**0.000**

TUKEY MULTIPLE COMPARISON : PROFILE

Level	A	B	MD	P
low	constant	pulse	-0.509	0.018
low	constant	cyclic	-1.387	**0.001**
low	cyclic	pulse	0.878	**0.001**
basal	constant	pulse	-0.036	0.900
basal	constant	cyclic	-0.592	**0.007**
basal	cyclic	pulse	0.556	0.012
high	constant	pulse	-0.198	0.661
high	constant	cyclic	-0.528	0.065
high	cyclic	pulse	0.330	0.337

TUKEY MULTIPLE COMPARISON : LEVEL

Profile	A	B	MD	P
constant	basal	low	-10.662	**0.001**
constant	basal	high	14.018	**0.001**
constant	high	low	-24.679	**0.001**
pulse	basal	low	-11.135	**0.001**
pulse	basal	high	13.857	**0.001**
pulse	high	low	-24.991	**0.001**
cyclic	basal	low	-11.456	**0.001**
cyclic	basal	high	14.082	**0.001**
cyclic	high	low	-25.539	**0.001**

tissue context

TWO-WAY ANOVA WITH INTERACTION

	SS	DF	MSE	F	P
**Profile**	8428.4	2	4214.2	952.0	**0.000**
**Level**	114499.5	2	57249.8	12932.8	**0.000**
**Interaction**	18162.6	4	4540.6	1025.7	**0.000**
Residual	1952.2	441	4.4	–	–
Total	143042.7	449	–	–	–

SIMPLE MAIN EFFECTS TESTING : PROFILE

Level	SS	DF	MSE	F	P
low	26548.2	2	13274.1	2998.6	**0.000**
basal	29.7	2	14.9	3.4	0.036
high	13.0	2	6.5	1.5	0.231

SIMPLE MAIN EFFECTS TESTING : LEVEL

Profile	SS	DF	MSE	F	P
constant	17570.5	2	8785.2	1984.6	**0.000**
pulse	25915.3	2	12957.6	2927.1	**0.000**
cyclic	89176.4	2	44588.2	10072.5	**0.000**

TUKEY MULTIPLE COMPARISON : PROFILE

Level	A	B	MD	P
low	constant	pulse	-6.212	**0.001**
low	constant	cyclic	-30.810	**0.001**
low	cyclic	pulse	24.598	**0.001**
basal	constant	pulse	-0.904	0.031
basal	constant	cyclic	0.075	0.900
basal	cyclic	pulse	-0.979	0.017
high	constant	pulse	-0.513	0.332
high	constant	cyclic	0.183	0.855
high	cyclic	pulse	-0.696	0.133

TUKEY MULTIPLE COMPARISON : LEVEL

Profile	A	B	MD	P
constant	basal	low	-11.950	**0.001**
constant	basal	high	14.519	**0.001**
constant	high	low	-26.469	**0.001**
pulse	basal	low	-17.257	**0.001**
pulse	basal	high	14.911	**0.001**
pulse	high	low	-32.168	**0.001**
cyclic	basal	low	-42.834	**0.001**
cyclic	basal	high	14.627	**0.001**
cyclic	high	low	-57.462	**0.001**

(**B**) Symmetry

colony context

TWO-WAY ANOVA WITH INTERACTION

	SS	DF	MSE	F	P
**Profile**	0.0	2	0.0	13.2	**0.000**
**Level**	0.2	2	0.1	161.1	**0.000**
**Interaction**	0.1	4	0.0	22.7	**0.000**
Residual	0.3	441	0.0	–	–
Total	0.7	449	–	–	–

SIMPLE MAIN EFFECTS TESTING : PROFILE

Level	SS	DF	MSE	F	P
low	0.1	2	0.0	57.6	**0.000**
basal	0.0	2	0.0	0.7	0.521
high	0.0	2	0.0	0.4	0.699

SIMPLE MAIN EFFECTS TESTING : LEVEL

Profile	SS	DF	MSE	F	P
constant	0.0	2	0.0	13.3	**0.000**
pulse	0.1	2	0.0	36.5	**0.000**
cyclic	0.2	2	0.1	156.7	**0.000**

TUKEY MULTIPLE COMPARISON : PROFILE

Level	A	B	MD	P
low	constant	pulse	-0.018	0.015
low	constant	cyclic	-0.058	**0.001**
low	cyclic	pulse	0.040	**0.001**
basal	constant	pulse	0.001	0.900
basal	constant	cyclic	0.006	0.539
basal	cyclic	pulse	-0.005	0.613
high	constant	pulse	-0.001	0.900
high	constant	cyclic	0.004	0.707
high	cyclic	pulse	-0.004	0.597

TUKEY MULTIPLE COMPARISON : LEVEL

Profile	A	B	MD	P
constant	basal	low	-0.014	0.021
constant	basal	high	0.014	0.025
constant	high	low	-0.028	**0.001**
pulse	basal	low	-0.033	**0.001**
pulse	basal	high	0.012	0.058
pulse	high	low	-0.045	**0.001**
cyclic	basal	low	-0.078	**0.001**
cyclic	basal	high	0.012	0.106
cyclic	high	low	-0.089	**0.001**

tissue context

TWO-WAY ANOVA WITH INTERACTION

	SS	DF	MSE	F	P
**Profile**	1.1	2	0.6	224.4	**0.000**
**Level**	2.3	2	1.2	477.6	**0.000**
**Interaction**	2.7	4	0.7	273.0	**0.000**
Residual	1.1	441	0.0	–	–
Total	7.2	449	–	–	–

SIMPLE MAIN EFFECTS TESTING : PROFILE

Level	SS	DF	MSE	F	P
low	3.8	2	1.9	766.7	**0.000**
basal	0.0	2	0.0	2.2	0.116
high	0.0	2	0.0	1.6	0.206

SIMPLE MAIN EFFECTS TESTING : LEVEL

Profile	SS	DF	MSE	F	P
constant	0.0	2	0.0	6.7	**0.001**
pulse	0.1	2	0.0	14.3	**0.000**
cyclic	4.9	2	2.5	1002.7	**0.000**

TUKEY MULTIPLE COMPARISON : PROFILE

Level	A	B	MD	P
low	constant	pulse	-0.021	0.336
low	constant	cyclic	-0.346	**0.001**
low	cyclic	pulse	0.325	**0.001**
basal	constant	pulse	-0.019	0.011
basal	constant	cyclic	-0.002	0.900
basal	cyclic	pulse	-0.017	0.028
high	constant	pulse	-0.001	0.900
high	constant	cyclic	0.015	**0.008**
high	cyclic	pulse	-0.016	**0.006**

TUKEY MULTIPLE COMPARISON : LEVEL

Profile	A	B	MD	P
constant	basal	low	-0.031	**0.001**
constant	basal	high	0.000	0.900
constant	high	low	-0.031	**0.001**
pulse	basal	low	-0.034	**0.001**
pulse	basal	high	0.018	0.042
pulse	high	low	-0.052	**0.001**
cyclic	basal	low	-0.375	**0.001**
cyclic	basal	high	0.017	0.447
cyclic	high	low	-0.393	**0.001**

(**C**) Cycle Length (hours)

colony context

TWO-WAY ANOVA WITH INTERACTION

	SS	DF	MSE	F	P
**Profile**	444.4	2	222.2	406.4	**0.000**
**Level**	2019.9	2	1009.9	1846.9	**0.000**
**Interaction**	1034.0	4	258.5	472.7	**0.000**
Residual	241.1	441	0.5	–	–
Total	3739.4	449	–	–	–

SIMPLE MAIN EFFECTS TESTING : PROFILE

Level	SS	DF	MSE	F	P
low	1474.9	2	737.4	1348.6	**0.000**
basal	0.6	2	0.3	0.5	0.584
high	2.9	2	1.5	2.7	0.069

SIMPLE MAIN EFFECTS TESTING : LEVEL

Profile	SS	DF	MSE	F	P
constant	1144.3	2	572.1	1046.3	**0.000**
pulse	1112.3	2	556.1	1017.0	**0.000**
cyclic	797.2	2	398.6	729.0	**0.000**

TUKEY MULTIPLE COMPARISON : PROFILE

Level	A	B	MD	P
low	constant	pulse	0.034	0.900
low	constant	cyclic	-6.635	**0.001**
low	cyclic	pulse	6.669	**0.001**
basal	constant	pulse	0.134	0.483
basal	constant	cyclic	0.132	0.489
basal	cyclic	pulse	0.001	0.900
high	constant	pulse	0.148	0.026
high	constant	cyclic	0.342	**0.001**
high	cyclic	pulse	-0.194	**0.002**

TUKEY MULTIPLE COMPARISON : LEVEL

Profile	A	B	MD	P
constant	basal	low	1.660	**0.001**
constant	basal	high	-4.850	**0.001**
constant	high	low	6.510	**0.001**
pulse	basal	low	1.561	**0.001**
pulse	basal	high	-4.836	**0.001**
pulse	high	low	6.396	**0.001**
cyclic	basal	low	-5.107	**0.001**
cyclic	basal	high	-4.641	**0.001**
cyclic	high	low	-0.466	**0.001**

tissue context

TWO-WAY ANOVA WITH INTERACTION

	SS	DF	MSE	F	P
**Profile**	1248.9	2	624.5	301.0	**0.000**
**Level**	1716.4	2	858.2	413.7	**0.000**
**Interaction**	2903.8	4	725.9	349.9	**0.000**
Residual	896.2	432	2.1	–	–
Total	6817.6	446	–	–	–

SIMPLE MAIN EFFECTS TESTING : PROFILE

Level	SS	DF	MSE	F	P
low	4177.4	2	2088.7	1006.9	**0.000**
basal	3.4	2	1.7	0.8	0.442
high	0.8	2	0.4	0.2	0.822

SIMPLE MAIN EFFECTS TESTING : LEVEL

Profile	SS	DF	MSE	F	P
constant	2985.5	2	1492.7	719.6	**0.000**
pulse	514.2	2	257.1	123.9	**0.000**
cyclic	1087.1	2	543.6	262.0	**0.000**

TUKEY MULTIPLE COMPARISON : PROFILE

Level	A	B	MD	P
low	constant	pulse	-8.965	**0.001**
low	constant	cyclic	-12.701	**0.001**
low	cyclic	pulse	3.736	**0.001**
basal	constant	pulse	0.257	0.030
basal	constant	cyclic	0.362	**0.001**
basal	cyclic	pulse	-0.105	0.547
high	constant	pulse	0.080	0.198
high	constant	cyclic	0.182	**0.001**
high	cyclic	pulse	-0.102	0.073

TUKEY MULTIPLE COMPARISON : LEVEL

Profile	A	B	MD	P
constant	basal	low	6.581	**0.001**
constant	basal	high	-4.385	**0.001**
constant	high	low	10.966	**0.001**
pulse	basal	low	-2.641	**0.001**
pulse	basal	high	-4.562	**0.001**
pulse	high	low	1.921	**0.001**
cyclic	basal	low	-6.482	**0.001**
cyclic	basal	high	-4.565	**0.001**
cyclic	high	low	-1.917	**0.001**
