# Supplementary material for: Model design choices impact biological insight: Unpacking the broad landscape of spatial-temporal model development decisions
Source: PLoS Comput Biol. 2024 Mar 8;20(3):e1011917. doi: 10.1371/journal.pcbi.1011917 (PMC10954156; doi:10.1371/journal.pcbi.1011917)
Supplement: Attachment — Submitted filename: PLOS23_ModelingChoices_Response.pdf [file pcbi.1011917.s016.pdf]

# Reviewer 1

*This manuscript reports the outcome of several computational experiments to test the effect of certain modeling choices on model behavior. The biological setting is tumor growth, modeling in an agent-based model framework. Features tested include choices of representation of tumor geometry, representation of cells, and spatial dimension. The results reported are that there are quantitative differences in metrics such as tumor growth or cell cycle length and, in some cases, qualitative differences.*

*When a modeler sets out to build a model of a biological system in order to understand it better and discover new features, then certain choices need to be made. They are determined by a number of considerations, such as whether quantitative or qualitative answers to questions are required, computational resources, degree of conformity to "reality," and others. A model can of course be wildly unrealistic and still provide valuable insights. The study reported here is intended to shed light on the effects of some choices to be made in the specific setting to be considered.*

*The reported results are qualitative, comparing one the outcome of one choice to another. It would be helpful in this case to compare both outcomes to "reality" in order to make an informed choice.*

Computational models are often trained to and validated against "real" experimental data. Assuming the reviewer is concerned about validation, we note that the purpose of this study is to demonstrate the importance of assessing modeling choices; we do not offer the reader specific guidelines/mandates, and we do not state that one modeling choice is superior/inferior to another. If we did, we agree that validation would be essential to this study. One modeling choice is not necessarily better than another; these decisions are highly context specific and not generalizable from one model system to another. We reinforce this point by adding additional language to the Discussion:

> It is important to note that the assessment of modeling choices is not a form of model validation and does not require experimental data.

*I do not find the reported results very useful, since it is not clear how they generalize to other biological settings. It is certainly not surprising that different choices of geometry, for instance, lead to differences in model behavior, but does it make a difference if I wanted to model a kidney disease rather than cancer?*

We agree that it isn't surprising that different modeling choices lead to different model behaviors. To determine if choices in geometry would, for example, lead to differences between a model of the kidney rather than a model of cancer, one would need to assess the impacts of the model decision on both models. However, such model decisions are seldom rigorously analyzed and reported, which compromise the utility and longevity of computational models.

The results reported in this manuscript are not intended to be generalizable; we believe that modeling choices are highly context specific and general guidelines would be misleading. Instead, we demonstrate the utility of interrogating modeling choices in complex multiscale models. The message we intend to relay is that "when you model A, depending on what you are interested in, it may be important to consider how your choice of X affects the outcomes". We do not intend to state "your choice of X will/will not make a difference when you model A vs. B".

To clarify our intent regarding generalizability, we modified the text in the Discussion:

> ...It is critical to make deliberate and informed choices when developing these models to effectively represent the system and address the question of interest. ~~We~~ Rigorous model development practices should include (and report) an assessment of the impact of modeling choices on the simulated behavior of interest. Similar to traditional model analyses (e.g., robustness analysis and sensitivity analysis), these assessments enable the modeler to better understand and refine both the structure and utility of their model for its designed purpose. In this study, we identified and interrogated how select modeling choices—at the system, cell and environment levels—impact observed temporal, spatial, and single-cell-level emergent behaviors in an ABM of cancerous cell growth. ... ~~These~~ Our modeling choices are agnostic to the specific modeling framework, and we are able to identify broadly applicable takeaways for ~~model development~~future models developed in this, or similar, contexts. However, given that all models are both question and context dependent, the conclusions derived from this study are not necessarily generalizable across all computational models; this reality further motivates the need to interrogate modeling choices as a necessary step in the formal model development process.

*As another example, knowing that 2D and 3D simulations tend to lead to different outcomes is useful, but knowing how to scale certain variables from a 2D simulation to a 3D one would be significantly more helpful.*

We agree that research on how to systematically scale variables from 2D to 3D would be useful and most exciting, but it is outside the scope of this study as the answer is not generalizable and can vary based on context, scale, resolution, etc. That being said, we expect our approach and assessment of model decisions to be fundamental to any researcher that aims to understand how the choice of characterizing a system in 2D or 3D would impact insights on their biological system or question of interest.

*In summary, I commend the authors for carrying out these studies. But any modeler should do the same before making these choices in order to build models that are credible for the intended purpose. However, the results reported are of limited use, as modeling platforms vary and different biological systems have different behavior leading to potentially quite different effects of model choices. For these reasons I cannot recommend publication of this manuscript.*

We certainly agree that modelers should evaluate the impact of their model design choices, but this evaluation is often not rigorously performed and a comprehensive evaluation of all possible design choices is impractical. We aim to demonstrate how a more focused evaluation of design choices can identify the conditions in which certain design choices do or do not impact the results.

In this study, we deliberately selected design choices that are commonly useful—representation of the system, agent variability, and environmental conditions—for a variety of systems. That is not to say that all of the choices we evaluated are applicable to all systems.

We revisited the language used in the manuscript and modified the narrative in order to add more context to the findings. Specifically, we clarify that "the choice of X vs. Y produces the following impact *in this system*" instead of "the choice of X vs. Y will produce the following impact" so it is clear that results are not necessarily generalizable and do not necessarily apply in all systems:

> . . . While choices in geometry and dimension produce qualitatively similar trends in this system; there are quantitative differences that are important to consider when developing models to address specific questions. . . .

> Overall, both initial cell volume and age variability seem to have more of an impact on cell-level and temporal trends than population-level and spatial trends, and their impacts are independent in this system. . . .

> . . . Overall, choices on nutrient dynamics have a significant impact on emergent outcomes in this system, particularly when the choices result in nutrient-limited conditions. . . .

# Reviewer 2

*Authors introduce an agent-based modeling approach which they use to study the effect of geometry, dimension, and context (colony/tissue) on some emergent behaviors (growth rate, symmetry, and cell cycle length). The results are explained within a broader discussion on modeling decisions. The results are presented in a coherent and systematic fashion, and the figures are very well designed. This is an important paper that investigates common agent-based decisions that every modeller must make.*

*My main (minor) criticism of the paper is that the Introduction is rather short, and I believe some of the description later in the paper would be better suited for the Introduction section. For example, the background info in the first three paragraph of the results may be better suited within the introduction. There reference to citation 12 on page 3 is a helpful guidance to the theme of the paper and authors should also consider moving this to the introduction.*

We appreciate the suggestion. We have moved the three paragraphs from the beginning of the Results section to the Introduction section and added a reference for additional background on agent-based models. We have not moved the citation the reviewer noted, as the scope of the citation is on experimentally observed similarities and differences between 2D vs. 3D, which is specific to the systems representation section of the results.

*There are several vague sentences in the results, describing that a change exists, but not the direction of the change (e.g. input parameter A causes emergent behavior B to rise/fall). Please clarify the following sentences w/ more concrete result:*

1. *Page 1, first paragraph: "...such models and results are not readily generalizable to other cell types or contexts." What context? Environmental context? Why is not generalizable? Some further description would be helpful.*
2. *Page 3, second paragraph: "Qualitative trends in emergent behavior across different choices of geometry and dimension are largely consistent over time" – what trends, and what exactly is consistent?*
3. *Page 8, second paragraph: "Tumor shape is strongly impacted by glucose level" – how does shape change? Or is it simply size?*

Thanks for bringing these points to our attention. We added the following sentences to the manuscript in order to clarify ambiguities in observed results.

For Page 1, first paragraph:

> For instance, a researcher may choose to include realistic mechanistic behavior for a specific cell type by developing a precise model that accurately captures a behavior of interest~~; such models and results are not readily~~ . However, analogous to overfit machine learning models that perform poorly on data outside its training set, a computational model developed and parameterized to represent a specific cell type within a specific environmental context may not be generalizable to other cell types or contexts.

For Page 3, second paragraph:

> ~~Qualitative trends in emergent behavior across~~ For different choices of geometry and dimension~~are largely consistent~~ , the trends in emergent behavior metrics are qualitatively similar over time.

For Page 8, second paragraph:

> Tumor ~~shape~~ size is strongly impacted by glucose level: higher glucose levels lead to larger tumors.

*Figure 5: there appears to be little effect of nutrient level (low/basal/high) in figure 5B. Why is that? This is not noted within the text.*

We updated the text to explain this observation:

> We note that the impact of glucose level is not clearly observed in nutrient concentrations over time due to the faster time scale of diffusion relative to the simulation time step (Figure 5B).

*There is an extensive literature on ABM's in math biology, which generally seems under-cited in the paper. Consider citing some of the following ABM reviews (or references contained therein):*

1. *Hybrid modeling frameworks of tumor development and treatment (PMID: 31313504)*
2. *Agent-based methods facilitate integrative science in cancer (PMID: 36404257)*
3. *Uncertainty and sensitivity analyses methods for agent-based mathematical models: An introductory review (Hamis et al)*
4. *A review of cell-based computational modeling in cancer biology (PMID: 30715927)*

We agree that ABMs have become increasingly popular and well-established in the field of computational biology. As described in PMID: 30715927, researchers actively use these ABMs to simulate highly detailed morphologies of few cells to competition among large diverse populations of simpler agents. While we have developed and continue to celebrate the use of ABMs in our own research, the purpose of this paper is not to "sell" the ABM framework. Instead, our focus is to bring attention to, and awareness of, how the analysis of modeling choices should be integrated into the model development process. Our use of ARCADE as the model testbed serves strictly as a vehicle to showcase this effort.

We have included a citation to the third review in the Discussion section in context of model analyses approaches and included the model used therein as an example of the "center slice" dimension choice:

> [13] Hamis S, Yates J, Chaplain MAJ, and Powathil GG. Targeting cellular DNA damage responses in cancer: An in vitro-calibrated agent-based model simulating monolayer and spheroid treatment responses to atr-inhibiting drugs. *Bulletin of Mathematical Biology*, **83**(10):103, October 2021. ISSN 0092-8240, 1522-9602. doi: 10.1007/s11538-021-00935-y.
>
> [19] Hamis S, Stratiev S, and Powathil GG. Uncertainty and sensitivity analyses methods for agent-based mathematical models: an introductory review. In *The Physics of Cancer*, pp. 1–37. World Scientific, December 2020. ISBN 9789811223488 9789811223495. doi:10.1142/9789811223495_0001.

# Reviewer 3

*This paper predominantly centers around the utilization of an ABM named ARCADE. However, there are several major concerns that have tempered my enthusiasm for the paper. These concerns are outlined below:*

*The title and abstract are misleading. Initially, I believed I was reading a review of modeling strategies rather than an exploration of a single modeling approach in a specific scenario. The title and abstract should be rephrased to accurately reflect the content presented in the paper.*

We modified the title to "Model design choices impact biological insight: Unpacking the broad landscape of spatial-temporal model development decisions" in order to highlight our focus on the evaluation of model design choices, rather than specific strategies therein. We also edited the abstract to further emphasize this point:

> ~~These generalizable insights~~ This generalizable investigation can help guide the choices made when developing biological models that aim to characterize spatial-temporal dynamics.

*The authors place significant emphasis on "emergent behavior." However, this term remains undefined, and none of the results seem to exhibit emergent behavior in the classical physical sense. Therefore, more precise and accurate terminology should be employed to describe the observed phenomena.*

We agree and we appreciate that different fields of study can use terminology in diverse ways. To alleviate ambiguity, we added a definition of emergence to the Introduction section:

> To elucidate the impact these design decisions have on model outcomes and inform future model design decisions, we investigate how common modeling choices influence resulting emergent behavior in context of an agent-based model (ABM). We define emergent behavior as a quantifiable property of any system that is not pre-defined and does not arise directly from parameters of constituent components.

*Numerous ABM platforms have been employed and published in various fields, ranging from cancer research to stem cell studies and beyond. It is unclear from this reading how the work presented relates to these other studies. The paper should clarify its contributions and distinctions in relation to existing research.*

ABM frameworks have indeed become increasingly popular and applied to a variety of biological systems. While this paper utilizes an ABM framework to perform the study, the approach and conclusions are intended to be broadly applicable across many different modeling frameworks. We have added several sentences to the Discussion section to clarify:

> ...It is critical to make deliberate and informed choices when developing these models to effectively represent the system and address the question of interest. ~~We~~ Rigorous model development practices should include (and report) an assessment of the impact of modeling choices on the simulated behavior of interest. Similar to traditional model analyses (e.g., robustness analysis and sensitivity analysis), these assessments enable the modeler to better understand and refine both the structure and utility of their model for its designed purpose. In this study, we identified and interrogated how select modeling choices—at the system, cell and environment levels—impact observed temporal, spatial, and single-cell-level emergent behaviors in an ABM of cancerous cell growth. ~~These~~ It is important to note that the assessment of modeling choices is not a form of model validation and does not require experimental data. Our modeling choices are agnostic to the specific modeling framework, and we are able to identify broadly applicable takeaways for ~~model development~~future models developed in this, or similar, contexts. However, given that all models are both question and context dependent, the conclusions derived from this study are not necessarily generalizable across all computational models; this reality further motivates the need to interrogate modeling choices as a necessary step in the formal model development process.

> ...Furthermore, additional emergent behavior metrics relevant to specific questions could be assessed. While ABM frameworks have become increasingly popular for characterizing biological systems, this evaluation can be applied to diverse model types to evaluate the impact of specific and relevant modeling choices.
